# Comparative analysis of bacterioplankton assemblages from two subtropical karst reservoirs of southwestern China with contrasting trophic status

**DOI:** 10.1038/s41598-020-78459-z

**Published:** 2020-12-18

**Authors:** Qiang Li, Yadan Huang, Shenglin Xin, Zhongyi Li

**Affiliations:** 1 Key Laboratory of Karst Dynamics, MNR and GZAR, Institute of Karst Geology, Chinese Academy of Geological Sciences, Guilin, 541004 China; 2 International Research Center on Karst Under the Auspices of UNESCO, Guilin, 541004 China; 3 Graduate School of Guilin Medical University, Guilin, 541004 China; 4 Agricultural Resource and Environment Research Institute, Guangxi Academy of Agricultural Sciences, Nanning, 530007 China

**Keywords:** Ecology, Microbial ecology

## Abstract

Although bacterioplankton play an important role in aquatic ecosystems, less is known about bacterioplankton assemblages from subtropical karst reservoirs of southwestern China with contrasting trophic status. Here, 16S rRNA gene next-generation sequencing coupled with water chemistry analysis was applied to compare the bacterioplankton communities from a light eutrophic reservoir, DL Reservoir, and a mesotrophic reservoir, WL Reservoir, in subtropical karst area of southwestern China. Our findings indicated that Proteobacteria, Firmicutes, Actinobacteria, Bacteroidetes, Cyanobacteria and Verrucomicrobia dominated bacterioplankton community with contrasting relative frequency in the two subtropical karst reservoirs. Proteobacteria and Bacteroidetes were the core communities, which played important roles in karst biogeochemical cycles. Though WT, TN and DOC play the decisive role in assembling karst aquatic bacterioplankton, trophic status exerted significantly negative direct effects on bacterioplankton community composition and alpha diversity. Due to contrasting trophic status in the two reservoirs, the dominant taxa such as *Enterobacter*, *Clostridium sensu stricto*, *Candidatus* Methylacidiphilum and *Flavobacteriia,* that harbor potential functions as valuable and natural indicators of karst water health status, differed in DL Reservoir and WL Reservoir.

## Introduction

The freshwater reservoir is a semi-artificial and semi-natural ecosystem providing water supply for domestic, industrial and agricultural use and represents an exclusive habitat for microbes performing critical functions in biogeochemical cycles^1^. However, under the impact of anthropogenic activities coupled with natural processes, organic/inorganic nutrients are gradually retained in freshwater reservoirs^2^. According to a report from United Nations Environment Programme, 40–50% of the lakes and reservoirs worldwide have been affected more or less by eutrophication^3^. Eutrophication not only influences water quality but also affects the composition, distribution and activity of aquatic bacterioplankton^4–6^.

To probe the response of bacterioplankton communities to trophic status in freshwater ecosystems in depth, Ji et al. found that eutrophic lakes were dominated by Cyanobacteria, yet oligotrophic lakes were dominated by Actinobacteria in Wuhan, China^7^. Iliev et al. pointed out that Proteobacteria, Actinobacteria and Bacteroidetes were the three dominant phyla in the oligotrophic reservoirs named Batak, Tsankov and Kamak in the Rhodope Mountains, Bulgaria^8^. In addition, Liu et al. revealed that cyanobacterial biomass cycle was strongly correlated with the community composition of eukaryotic plankton in the subtropical reservoirs from southeast China^9^. However, a confusing problem relating to aquatic bacterioplankton ecology has not been clarified about which environmental factor plays a decisive role in shaping bacterioplankton communities^5,9^.

Across the karst regions representing 7–12% of the Earth’s continental area, about 25% of the global population is supplied by karst waters^10^. Under the influence of carbonate rock weathering in karst, karst water is characterized by high concentrations of Ca^2+^ and HCO_3_^−^, where calcium mediates stabilisation of organic matter and acts as a determining factor in shaping bacterial populations and activity^11^. Moreover, strong linear relationships exist between EC versus Ca^2+^ and HCO_3_^–^^12^. Thus, Ca^2+^, HCO_3_^−^ and EC can be grouped to indicate karst level of karst waters. Besides that, the equilibrium (CaCO_3_ + H_2_O + CO_2_ ⇄ Ca^2+^ + 2HCO_3_^−^) and DO are influenced by WT, which in turn affects pH^11,12^. Consequently, WT, DO and pH are grouped as hydrochemical factors in karst waters. Though previous findings provided insights into the diversity and dynamics of microbes in karst springs^13^, unsaturated and saturated karst aquifers^14^, water pools^15^, groundwater-surface water exchange systems^16^ and karst dammed rivers^11^, less attention has been paid to the comparative analysis of bacterioplankton assemblages from subtropical karst reservoirs of southwestern China, especially with contrasting trophic status. Considering that building reservoirs is the formidable engineering challenges in karst^17^, it is not easy to find karst reservoirs with contrasting trophic status at adjacent areas. Basing on our early investigation^18,19^, DL Reservoir (a light eutrophic reservoir) and WL Reservoir (a mesotrophic reservoir) with distance in a straight line of 314 km were selected in our study to explore the influence of trophic status on bacterioplankton assemblages from karst reservoirs under the same climate background. Consequently, two questions are addressed in our study: (i) Does karst level influence trophic status? and (ii) are karst aquatic bacterioplankton assemblages with contrasting trophic status similar or not? To answer the above questions, aquatic bacterioplankton were sampled at the same season from different depths and examined by using 16S rRNA gene next-generation sequencing. Moreover, water chemistry was analyzed. Thus, the obtained data will improve our fundamental understanding of the comparison results from bacterioplankton assemblages with contrasting trophic status in subtropical karst reservoirs.

## Materials and methods

### Study sites

**Figure 1:**
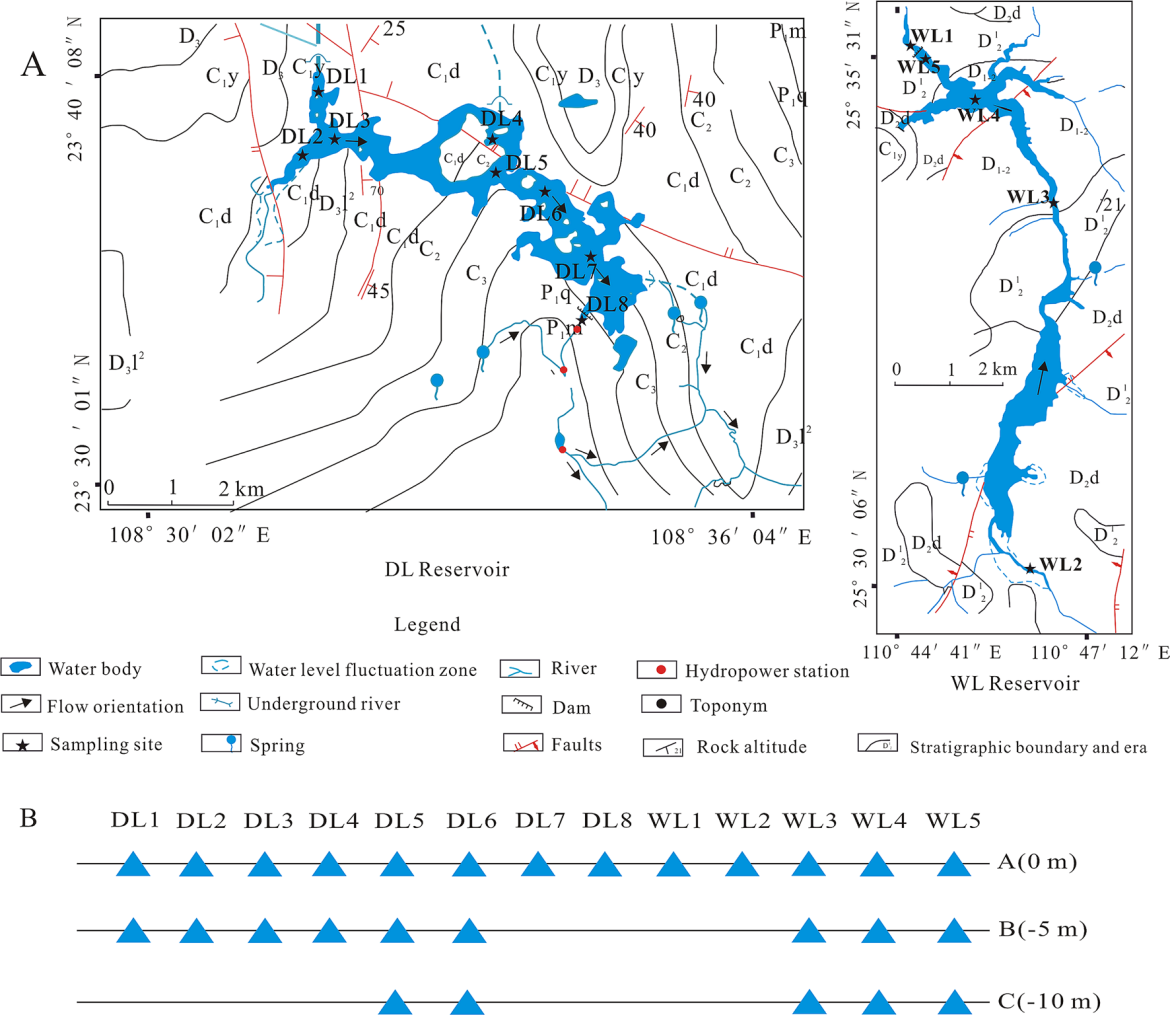
Sampling sites (**A**) and changes with water depth (**B**) in WL Reservoir and DL Reservoir.

The study sites were two reservoirs in GZAR of southwestern China, DL Reservoir (area: 8.05 km^2^, reservoir storage capacity: 1.09 × 10^9^ m^3^, 23° 30′ 1″–23° 40′ 8″ N, 108° 30′ 2″–108° 36′ 4″ E, depth: from − 4 to − 12 m), which is located in Shanglin County, GZAR, and replenished by an underground river, and WL Reservoir (area: 2.81 km^2^, reservoir storage capacity: 1.08 × 10^9^ m^3^, 25° 30′ 06″–23° 35′ 31″ N, 110° 44′ 41″–110° 47′ 12″ E, depth: from − 4 to − 12 m), which is located in Xing’an County, GZAR, and replenished by a land-surface river (Fig. 1, which was generated by QGIS with version 3.16.0 (https://www.qgis.org/en/site/)). Both reservoirs located in subtropical carbonate rock areas have been used as storage reservoirs for hydropower production and irrigation. Both reservoirs also have been used in previous research on hydro-bio-geo-chemical processes and were described in more detail in Lu et al.^18^ and Xin et al.^19^, where both reservoirs with contrasting Ca^2+^ and HCO_3_^−^ concentrations are Ca^2+^-HCO_3_^−^ type water. Moreover, the two reservoirs all stopped fishing in 2013.

### Sampling

According to USEPA TMDL guidelines^20^, three sampling zones were designed in every reservoir from upstream to downstream. If water depth is less than 5 m, sampling layer is set at surface. If water depth is less than 10 m, sampling layers are set at surface and − 5 m. If water depth is more than 10 m, sampling layers are set at surface, − 5 m and − 10 m. Thus, 27 samples were taken from DL Reservoir and WL Reservoir in August 2017 (Fig. 1). Eight sampling sites were selected in DL Reservoir from upstream zone (DL1, DL2 and DL3), midstream zone (DL4, DL5 and DL6) and downstream zone (DL7 and DL8). Five sampling sites were selected in WL Reservoir from upstream zone (WL2), midstream zone (WL3 and WL4) and downstream zone (WL1 and WL5). Samples were named according to the sampling sites (such as WL1) and specific depth (A: surface water, B: − 5 m and C: − 10 m), in that particular order (e.g., WL1A).

A water sample (approximately 3 L) for bacterioplankton DNA extraction was pre-filtered in situ by using a 3 µm Millipore GSWP membrane and then filtered through a 0.22 µm Millipore GSWP membrane according to the methods described by Li et al.^16^. All bacterioplankton samples on the 0.22 µm membranes were stored at − 80 °C until further processing.

WT, DO, pH, TDS and EC were measured in situ by an EXO Multiparameter Sonde (YSI, USA). HCO_3_^−^ and Ca^2+^ concentrations were titrated in situ by using an alkalinity test and a calcium test (Merck KGaA, Germany). Water samples for laboratory analysis were immediately transferred into high-density polyethylene bottles, with a volume of 1500 mL. Cations and TP were determined by an IRIS Intrepid II XSP full-spectrum direct-reading plasma spectrometer (Thermo Fisher Scientific, USA), anions were determined by MIC ion chromatography (WanTong, Switzerland), and TOC, DOC and TN were determined by a multi-N/C 3100 carbon–nitrogen analyzer (Jena, Germany). TSI is an effective way to evaluate the trophic status of a reservoir^21^. Though Chl a is an important parameter to determine the trophic status, the focus on Chl a as a biomass indicator would underestimate the final value of the TSI^21^. There is evidence that TSI (that is, Chl a) value indicates oligotrophy in 18 tropical/subtropical reservoirs with the max Chl a of 1804 µg/L, that makes questionable accuracy of the TSI index^21^. In this respect, Chl a, SD depth, TP, TN and COD_Mn_ were used in our study to calculate TSI (for eutrophic water, TSI > 50; for mesotrophic water, 30 < TSI < 50)^21,22^. Then, water transparency conditions were determined by SD, COD_Mn_ was determined by the potassium permanganate acid method, and Chl a was determined according to Lorenzen^22^.

### DNA extraction and sequencing of 16S rRNA genes

Bacterioplankton DNA was extracted from the filter membranes stored at − 80 °C using FastDNA Spin Kit for Soil (MP Biomedicals, USA) following the manufacturer’s instructions. The DNA concentration and quality were determined by Quawell Q5000 (Quawell, USA). After that, good-quality DNA was amplified for the V4–V5 region of 16S rRNA genes by using the PCR primers 515F (GTGYCAGCMGCCGCGGTA) and 909R (CCCCGYCAATTCMTTTRAGT)^23^. The PCR products amplifying the V4–V5 region of 16S rRNA genes were sequenced via the Illumina MiSeq platform (Illumina, USA) at the Chengdu Institute of Biology, Chinese Academy of Sciences.

### Bioinformatics analysis

In our article, fast length adjustment of short reads software was used to merge and extend the paired-end Illumina reads using the default parameters, with a maximum overlap of 400 bp. Moreover, low-quality reads with lengths below 200 bp and average quality scores below 30 were excluded. After that, the raw sequence data were processed using QIIME 1.7.0 software^24^. During the process, sequences matching plant chloroplast or mitochondrial 16S rRNA were filtered. Representative sequences from each OTU clustered at the 97% similarity level were aligned with the PyNAST aligner to the SILVA128 database of bacterial taxonomy. 829,931 high-quality and chimera-free reads with an average length of 425 bp grouped into 1292 OTUs from 27 water samples were generated. Sequences were aligned using the Aware Infernal Aligner in the RDP pyrosequencing pipeline, and subjected to chimera check using the Uchime algorithm, and resampling to 30,738 sequences per sample. Bootstrap OTU richness, Chao1 and ACE estimates, inverse Simpson index (a measure of evenness) and Simpson diversity index were calculated on rarefied OTU tables to assess the distribution patterns of bacterioplankton communities’ OTUs. Beta diversity was measured using Bray–Curtis dissimilarity coefficients. In addition, the Goods coverage ranged from 96.46 to 98.76%.

### Statistical analysis

All statistical analyses were carried out with R 3.6.1 for Windows (www.r-project.org). A Venn diagram was generated to evaluate the difference of bacterioplankton communities in WL Reservoir and DL Reservoir. PCoA plots based on Bray–Curtis distance were generated to determine the bacterioplankton community dissimilarity in all samples^25^. ANOSIM was carried out to determine the dissimilarity between WL Reservoir and DL Reservoir based on Bray–Curtis distances, where ANOSIM R varies between 0 and 1, with 0 representing no differences, and 1 representing complete difference^26^. RDA is a direct gradient analysis extension of principal components analysis and is a form of multivariate regression^27^. Then, RDA was performed to detect the strength of water physical–chemical parameters upon bacterioplankton community structure with all samples. Heat maps were generated to illustrate the relative frequencies of the most abundant OTUs in all samples and the relationships between the most abundant OTUs and physical–chemical variables. Correlation networks were visualized and customized using Gephi 0.9.2 to detect the interactions among the most abundant OTUs and water physical–chemical variables, and explore co-occurrence patterns in bacterioplankton communities with *P* values < 0.05 based on Pearson’s product-moment correlation^28^. Moreover, correlation analyses with a two-tailed probability were performed using the Pearson correlation method. The PM test based on Pearson's product-moment correlation was applied to explore the correlations among bacterioplankton communities (main phyla with relative abundance > 0.1%), alpha diversity, karst level (Ca^2+^, HCO_3_^−^ and EC), trophic status (TN, TP, TOC, DOC, COD_Mn_ and Chl a) and hydrochemical factors (WT, pH and DO) using PASSaGE 2 to eliminate collinearity between variables^29^. PLS-PM was used to explore the estimates of path coefficients (representing the direction and strength of the linear relationships between bacterioplankton communities, alpha diversity, karst level, trophic status and hydrochemical factors) and explained variability (*R*^2^)^29^. Moreover, VPA was performed to quantify the relative contributions of karst level, trophic status and hydrochemical factors to bacterioplankton community^29^.

## Results

### Water physical–chemical characteristics of the two reservoirs

**Figure 2:**
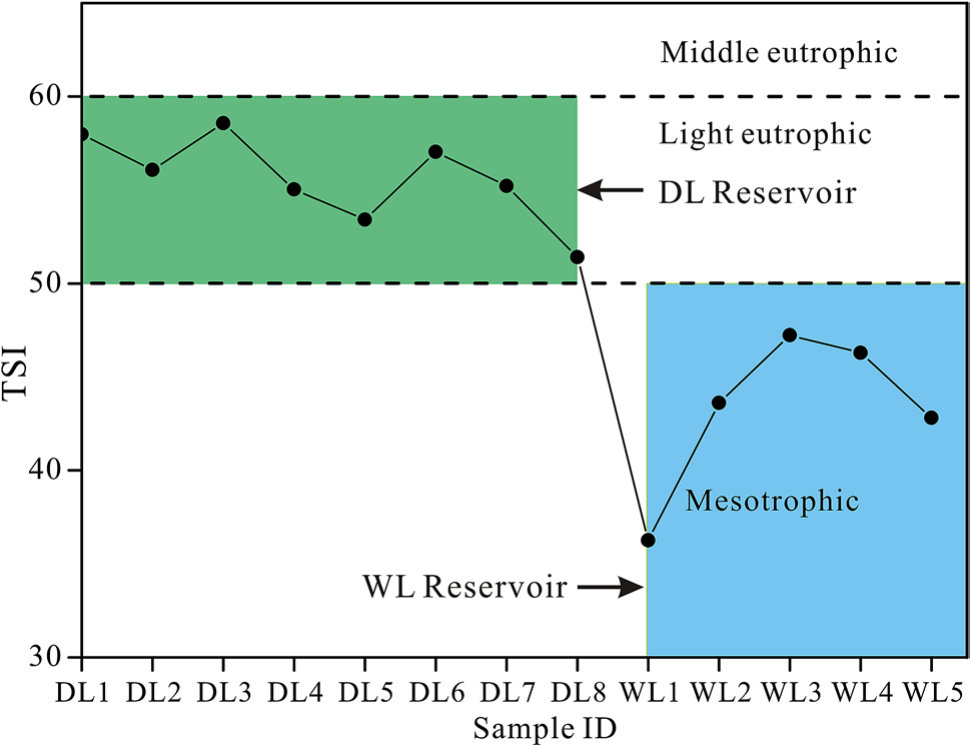
TSI values of WL Reservoir and DL Reservoir.

Water physical–chemical characteristics and TSI of both reservoirs are listed in Table 1 and shown in Fig. 2. The two reservoirs had high Ca^2+^ and HCO_3_^−^ concentrations as well as EC. HCO_3_^−^ accounted for 92.84% and 89.04% of the total anions in DL Reservoir and WL Reservoir, respectively. Moreover, the concentration of Ca^2+^ in DL Reservoir was three times higher than that in WL Reservoir. Figure 2 shows that TSI values in DL Reservoir ranged from 51.41 to 58.56 and that TSI values in WL Reservoir ranged from 36.25 to 47.24. Besides that, the values of Chl a, TOC, TN, TP, TDS and COD_Mn_ in DL Reservoir were higher than those in WL Reservoir.

**Table 1:** Physical–chemical characteristics of water samples from WL Reservoirs and DL Reservoir.

Sample ID	WT (°C)	pH	EC (µs/cm)	Ca^2+^ (mg/L)	HCO_3_^−^ (mg/L)	DO (mg/L)	Chl a (µg/L)	TOC (mg/L)	TN (mg/L)	TP (mg/L)	COD_Mn_ (mg/L)	TDS (mg/L)
DL1A	31.50	8.21	219.30	40.00	140.30	10.50	52.08	8.72	2.84	0.05	0.94	110.70
DL1B	25.02	7.13	337.00	62.00	213.50	5.60	6.83	4.71	3.05	0.01	0.59	170.00
DL2A	35.87	8.43	183.40	30.00	91.50	7.10	55.24	7.90	2.83	0.03	1.00	92.53
DL2B	25.32	7.19	334.00	72.00	225.70	3.74	6.96	5.09	2.95	0.01	0.52	173.70
DL3A	34.46	8.89	181.40	32.00	85.40	8.98	65.90	8.41	3.04	0.04	1.11	91.61
DL3B	25.14	7.17	363.70	74.00	244.00	3.60	7.17	5.19	3.00	0.01	< 0.50	183.50
DL4A	32.77	8.61	173.00	31.00	85.40	7.54	71.85	8.88	3.14	0.01	0.87	87.30
DL4B	27.61	7.36	294.70	60.00	183.00	4.26	37.44	5.34	2.93	0.02	0.52	148.00
DL5A	33.03	8.58	182.30	32.00	128.10	7.35	42.87	9.07	2.91	0.01	0.87	92.00
DL5B	27.59	7.26	289.40	60.00	195.20	4.64	35.70	5.89	2.86	0.03	0.59	145.00
DL5C	25.11	7.22	253.70	78.00	244.00	3.88	13.85	4.11	1.59	0.03	< 0.50	178.50
DL6A	32.20	8.84	175.10	27.00	115.90	9.57	44.19	8.76	2.81	0.02	1.00	88.39
DL6B	27.55	7.30	287.20	59.00	195.20	4.88	42.55	5.73	2.79	0.02	0.55	145.10
DL6C	25.45	7.34	345.00	70.00	225.70	4.19	20.18	4.68	1.59	0.01	< 0.50	174.10
DL7A	34.07	8.74	166.60	27.00	97.60	8.19	61.81	8.17	3.03	0.03	0.94	84.19
DL8A	26.42	7.36	314.50	68.00	274.50	4.81	24.11	5.13	2.94	0.02	0.52	158.80
WL1A	25.67	7.18	98.97	20.00	61.00	3.42	0.68	1.66	0.85	0.01	< 0.50	49.94
WL2A	26.23	8.81	101.70	21.00	36.60	2.95	1.35	1.70	1.30	0.01	< 0.50	51.35
WL3A	28.95	8.56	92.75	16.00	42.70	7.30	8.86	1.92	1.29	0.01	< 0.50	46.83
WL3B	28.57	8.55	94.05	19.00	61.00	6.95	8.45	1.79	1.13	0.01	0.81	47.49
WL3C	27.79	7.99	102.30	20.00	67.10	6.44	2.93	1.72	0.92	0.01	< 0.50	51.67
WL4A	29.60	8.44	79.50	14.00	48.80	7.43	7.65	1.87	0.97	0.01	< 0.50	40.13
WL4B	28.66	7.47	90.34	16.00	54.90	5.72	3.31	1.70	0.96	0.01	< 0.50	45.61
WL4C	27.53	7.26	102.60	18.00	61.00	4.73	1.62	1.66	1.03	0.014	< 0.50	51.78
WL5A	29.49	8.29	76.96	14.00	39.65	6.69	6.03	1.90	1.01	0.01	< 0.50	39.26
WL5B	27.67	7.37	87.36	15.00	48.80	5.63	3.18	1.77	1.00	0.09	< 0.50	44.09
WL5C	27.43	7.07	96.93	18.00	61.00	3.93	1.51	1.52	1.07	0.01	< 0.50	48.91

### Abundance and diversity of bacterioplankton communities

**Figure 3:**
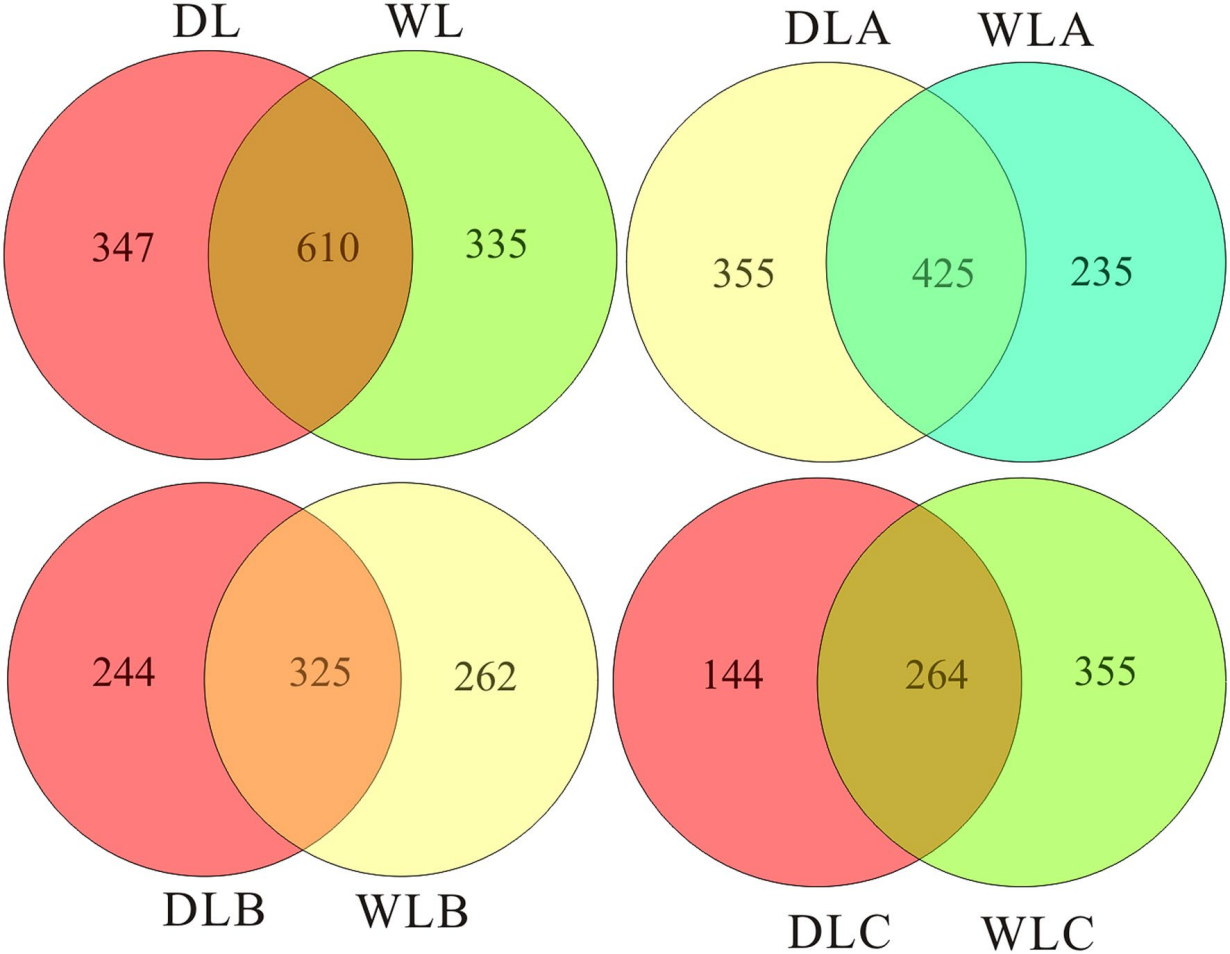
A Venn diagram with shared and unique OTUs of WL Reservoir and DL Reservoir.

A Venn diagram with shared and unique OTUs showed the difference of bacterioplankton from the two reservoirs (Fig. 3). The number of the total observed OTUs in the two reservoirs was 1292, with 610 OTUs (approximately 47.21% of the total) shared by them. The majority (62.95%) of the shared OTUs were Proteobacteria and Bacteroidetes. The percentage of unique OTUs in DL Reservoir and WL Reservoir was 52.79%, while they contributed to 36.26% and 35.45% of their own community genetic information, respectively. Besides, the shared and unique OTUs presented the difference with water depth changes. That is, the proportion of OTUs unique to DL Reservoir decreased with depth; however, the ratio of unique OTUs versus the total observed OTUs in WL Reservoir increased with depth.

**Figure 4:**
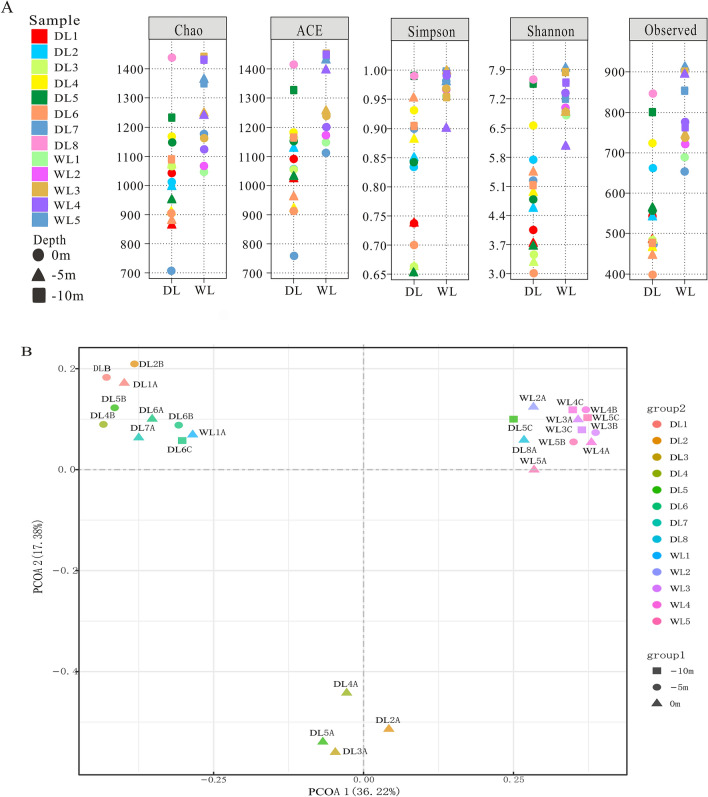
Alpha diversity of bacterioplankton communities from WL Reservoir and DL Reservoir (**A**) and PCoA plots representing bacterioplankton community dissimilarity of all samples based on the Bray–Curtis distances (**B**).

The alpha and beta diversity indices examined the difference of bacterioplankton communities in DL Reservoir and WL Reservoir (Fig. 4). It was found that alpha diversity indices in WL Reservoir were higher than those in DL Reservoir (Fig. 4A). Besides, higher alpha diversity indices in DL Reservoir were found in surface samples than those in subsurface samples, except for DL5C. In contrast, the highest alpha diversity indices in WL Reservoir were found in deep samples. The beta diversity analysis based on Bray–Curtis distances further illustrated the difference between DL Reservoir and WL Reservoir (Fig. 4B). Though highly similar communities in each reservoir were observed, every sample had a different and native bacterioplankton community, where the microenvironment was different. The significant dissimilarity between DL Reservoir and WL Reservoir were also supported by ANOSIM results based on Bray–Curtis distances (Fig. S1) and co-occurrence patterns (Fig. S2).

### Bacterioplankton community structure

**Figure 5:**
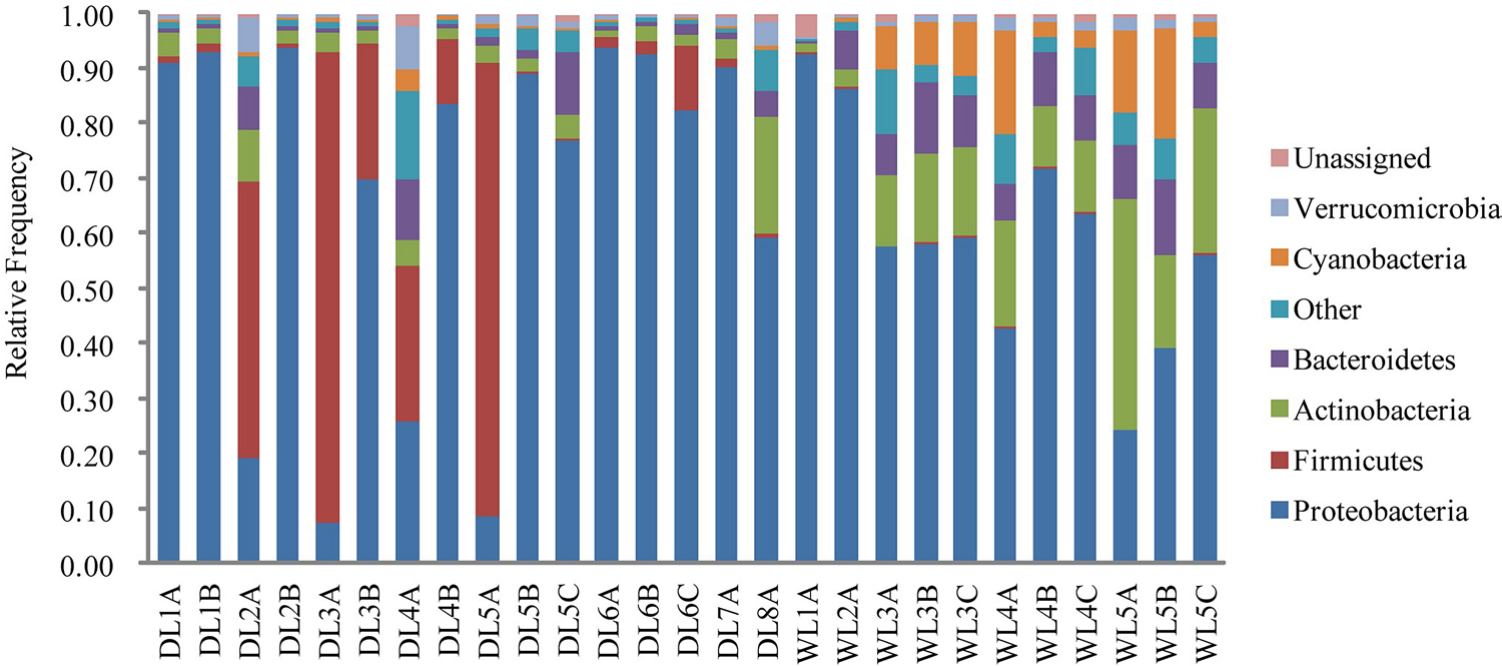
The phyla relative abundances of bacterioplankton communities in WL Reservoir and DL Reservoir. Phyla with relative abundances < 0.1% in all samples were grouped as ‘other phyla’.

**Figure 6:**
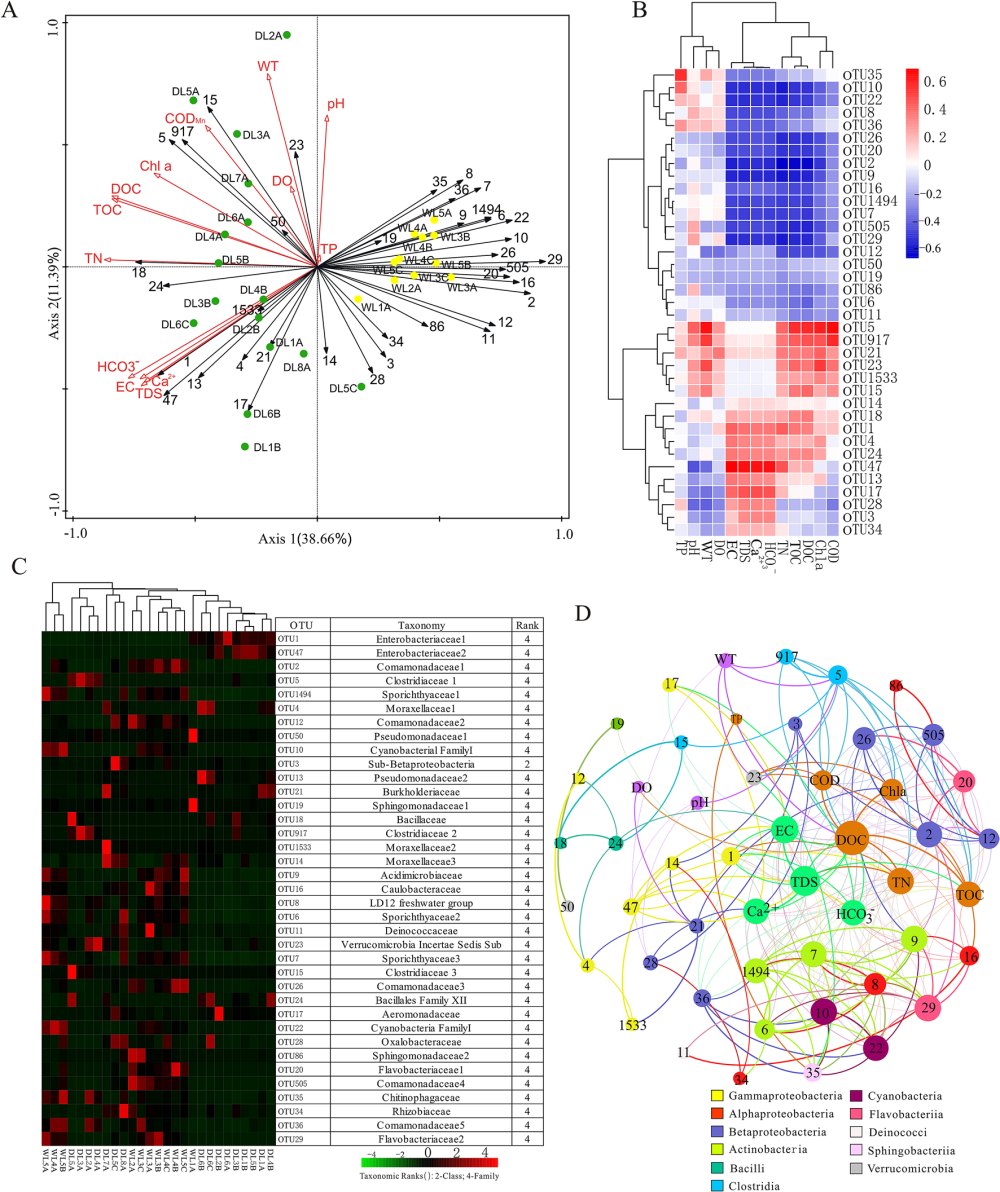
RDA plot indicating the relationship between the 37 most abundant OTUs and physical–chemical factors (green dots represent the samples from DL Reservoir, and yellow dots represent the samples from WL Reservoir) (**A**). Heat map illustrating the relationship between physical–chemical variables and the 37 most abundant OTUs (**B**). Heat map illustrating the relative frequency of the 37 most abundant OTUs in WL Reservoir and DL Reservoir (**C**). Correlation network of significant correlations among OTUs (node color corresponds to taxonomic affiliation) and between OTUs and physical–chemical variables (**D**). Node size is proportional to the OTU’s relative frequency.

All of the reads, specifically 95.66%, were assigned to 27 phyla; the remainder had not been previously classified at the phylum level. The variations of bacterioplankton community compositions were evident between DL Reservoir and WL Reservoir with the contribution of depth (Fig. 5). Proteobacteria, Firmicutes, Actinobacteria, Bacteroidetes, Cyanobacteria and Verrucomicrobia with contrasting relative frequency dominated bacterioplankton communities of the two reservoirs, which accounted for 63.92%, 11.45%, 9.24%, 5.24%, 3.73% and 1.62% of the total reads (mean relative frequency > 1%), respectively. Moreover, the distribution of the 37 most abundant OTUs reflected the prominent changes in all samples from DL Reservoir and WL Reservoir (Fig. 6C).

### Relationship between bacterioplankton communities and physical–chemical factors

The RDA plot revealed that the 37 most abundant OTUs distribution across all samples could be explained by the RDA1 axis (*P* = 0.002), significantly correlating with WT (*P* = 0.002), TN (*P* = 0.002) and DOC (*P* = 0.022), and grouped into two clusters (that is, two reservoirs) (Fig. 6A). The physical–chemical factors explained 68.8% of bacterioplankton variance, with axis 1 explaining 38.66% of the variance and axis 2 explaining 11.9%. Moreover, the association among the 37 most abundant OTUs in all samples and physical–chemical factors showed that the physical–chemical factors had negative or positive correlations with the 37 most abundant OTUs (Fig. 6B). Network analysis further showed the associations between co-occurring OTUs with physical–chemical factors (Fig. 6D).

**Figure 7:**
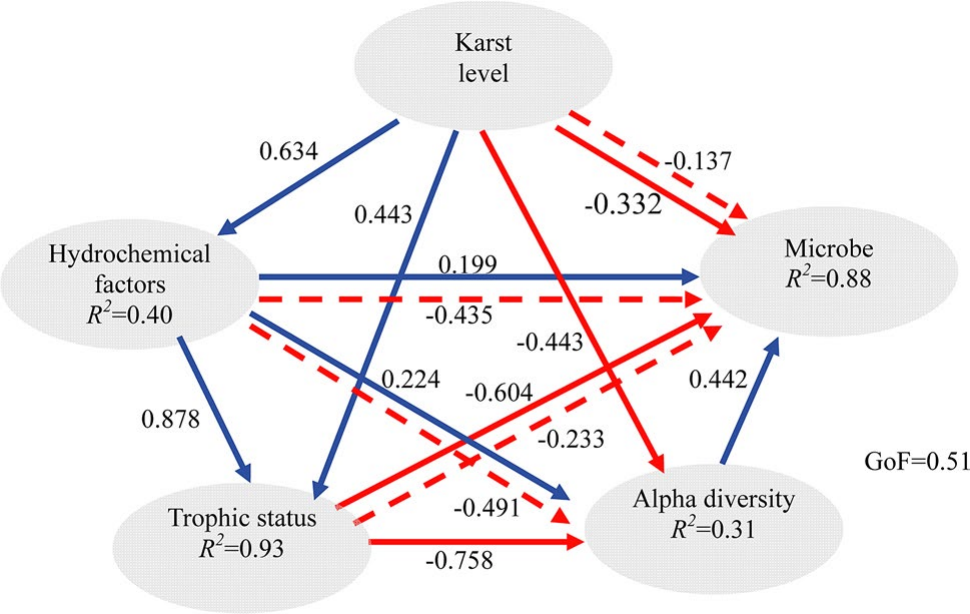
Directed graph of the PLS-PM of karst level, trophic status and hydrochemical factors on bacterioplankton communities. Path coefficients were calculated after 999 bootstraps, blue and red indicate positive and negative effects, respectively, and the solid and dashed lines indicate direct and indirect effects, respectively. The model was assessed using the GoF statistic, and the GoF value was 0.51.

PM test indicated that karst level, trophic status and hydrochemical factors had significant effects on bacterioplankton community (*P* < 0.05) (Table 2). To better integrate the complex interrelationships among bacterioplankton community and environmental factors, a PLS-PM represented here with GoF 0.51 was constructed (Fig. 7). The resulting PLS-PM indicated that karst level exerted significant direct effects on hydrochemical factors, hydrochemical factors exerted significant direct effects on trophic status, and there was a significantly negative direct effect of trophic status on bacterioplankton community and alpha diversity. These results were also confirmed by VPA analysis (Fig. S3). Trophic status alone explained 11% (*P* = 0.001) of the bacterioplankton community variation, while hydrochemical factors and karst level alone explained small portions of the observed variation, which accounted for 0 (*P* = 0.416) and 1% (*P* = 0.15), respectively. However, trophic status, karst level and hydrochemical factors explained weak portions of the observed variation about alpha diversity (*P* > 0.4). Taken together, our results indicated that trophic status played the important role in assembling bacterioplankton community from karst reservoirs.

**Table 2:** The influences of karst level, trophic status and hydrochemical factors on bacterioplankton communities by partial Mantel test.

Effect of	a	b	c	a	a	b	b	c	c	a	b	c
Controlling for				b	c	a	c	a	b	b + c	a + c	a + b
Bacterioplankton communities	*r*	*r*	*r*	*r*	*r*	*r*	*r*	*r*	*r*	*r*	*r*	*r*
	**0.393**	**0.443**	**0.159**	**0.308**	**0.377**	**0.373**	**0.421**	0.103	− 0.048	**0.311**	**0.365**	− 0.064

Bold indicates a significant correlation (*P* < 0.05).

^a^Karst level includes Ca^2+^, HCO_3_^−^ and EC.

^b^Trophic status includes TN, TP, TOC, DOC, COD_Mn_ and Chl a.

^c^Hydrochemical factors includes WT, pH and DO.

## Discussion

### Physical–chemical characteristics in the two reservoirs

In carbonate rock area, precipitation or dissolution of CaCO_3_ are affected by temperature, precipitation and CO_2_ concentration^12^. When water flows through karst conduits, carbonate rocks will be dissolved as a result of the water–carbonate rock–CO_2_ gas interactions^12^. Karstification degree (that is, karst level) in DL Reservoir replenished by an underground river was higher than that in WL Reservoir replenished by a land-surface river, which may have been due to the compound influence of replenished water source, carbonate dissolution processes and host rock^30^. Considering the chelating effect of calcium on other ions or chemical compounds^31^, the detention time of organic/inorganic nutrients in karst aquatic ecosystems will be affected by Ca^2+^ and HCO_3_^−^ concentrations, which in turn affects the trophic status of karst water. In the present study, it was found that DL Reservoir was a light eutrophic reservoir, and WL Reservoir was a mesotrophic reservoir. Then, our results suggest that karst level could affect trophic status and other hydrochemical factors.

### Taxonomy and distribution of bacterioplankton in the two reservoirs

In our study, the dominant phyla had different relative frequencies in their habitats due to them inhabiting different niches^32^. Moreover, the difference of microenvironment in the two reservoirs resulted in the negative or positive interrelationships (i.e., competitive or cooperative interactions) among the bacterioplankton^33^. As expected, the interaction networks became less complex over trophic status, suggesting that competition for light causes bacterioplankton biodiversity loss after eutrophication^32^.

The influence of physical–chemical characteristics on the most frequent OTUs that can be classified at the genus level in the two reservoirs was explored. It was found that *Sphingomonas* (OTU 19) and *Novosphingobium* (OTU 86) affiliated with Alphaproteobacteria dominated the surface water of mesotrophic WL Reservoir due to their adapting to oligotrophic environments, especially at the depth of − 030 to − 120 cm^34^. The most abundant Gammaproteobacteria-related OTUs (1, 4, 13, 14, 17, 50 and 1533) dominating in light eutrophic DL Reservoir (especially in the deep layer) may be relating to these freshwater tribes that were not suited to residence in the upper aquatic layer^32^. Considering that Oxytetracycline is one of the most used antibiotics in aquaculture, *Pseudomonas* (OTU13 and 50), the oxytetracycline-resistant species in freshwater, comprised a moderate fraction in the two reservoirs, which may be due to oxytetracycline remaining in the karst aquatic environment for a long time after cessation of use^35^. The *Variovorax* (OTUs 12 and 505) and *Simplicispira* (OTU36) that were affiliated with Betaproteobacteria and aerobic motile organisms dominated in WL Reservoir, reflecting the difference of oxygen supply with contrasting trophic status^36,37^. Moreover, the minute photosynthetic prokaryote *Prochlorococcus* (OTUs 10, 22 and 33) that were affiliated with Cyanobacteria were dominant in WL Reservoir, suggesting that they could supply oxygen for *Variovorax* and *Simplicispira*^32^. The genus *Clostridium sensu stricto* (OTUs 5 and 917) affiliated with Firmicutes, the minor freshwater lake phylum, were dominant in DL Reservoir, especially in DL2A, DL3A, DL4A and DL5A, though few studies have reported their presence, distribution, or activity in the epilimnion of freshwater lakes^32^. They also had an important role in the degradation of insoluble organic pollutants, such as petroleum hydrocarbons^38^ and polychlorinated biphenyl^39^, suggesting that these organic pollutants might be from the recharge area influenced by agricultural activity. However, further studies are still needed to understand the role of *Clostridium sensu stricto* in the karst reservoir. The OTUs 18 (*Bacillus*) and 24 (*Exiguobacterium*) affiliated with Firmicutes dominating the deep layer of DL Reservoir may be relating to their inhibiting effect on eukaryotic and other prokaryotic autotrophs, including Cyanobacteria^40,41^. The most abundant Verrucomicrobia-related OTU 23 (*Candidatus* Methylacidiphilum) in DL Reservoir has been reported as a thermoacidophilic methanotroph in tropical and summer temperate lakes^32^, which was associated with autochthonous carbon production and able to conduct assimilatory nitrate reduction and reduce NO_2_^−^–N to N_2_O, suggesting their important role in carbon and nitrogen cycles^5,42^. Considering that *Flavobacteriia* affiliated with Bacteroidetes could be a valuable natural indicator of “system disturbances” in karst aquifers and was favoring in less eutrophic lakes^15,32,43^, it was found that *Flavobacterium* (OTU 29) and *Cloacibacterium* (OTU 20) were mainly detected in WL Reservoir. The actinobacterial hgcI clade (OTUs 6, 7 and 1494) affiliated with Actinobacteria were dominant in WL Reservoir, which may be due to their adaptation to nutrient-poor environments^5^. In contrast, the presence of the CL500-29 marine group affiliated with Actinobacteria dominating in WL Reservoir was surprising, yet a better understanding of their roles and the influence of geological background in karst freshwater is still needed in the future^11^.

### Physical–chemical factors shaping bacterioplankton communities

Many previous studies showed that bacterioplankton communities were shaped by their habitats^32^. In our study, Venn analyses revealed that Proteobacteria and Bacteroidetes were the core communities in our study, which has been reported by Shabarova et al. in karst aquifers^15^. The majority of the shared OTUs in all samples were also consistent with other studies describing the ‘core OTUs’ in flooded subsurface karst water pools^15^, which highlighted the importance of environmental forces^44^. It was well known that TDSs mainly influenced by high concentrations of calcium might reveal the nutritional levels of the water^45^. In our study, DL Reservoir may represent one extreme of TDSs increasing along depth with high inorganic and organic content, and WL Reservoir may represent one extreme of TDSs decreasing along depth with relatively low concentrations of inorganic and organic matter, leading to a high proportion of unique bacteria important for biogeochemical cycles in the deep layer of DL Reservoir. In any case, the increased nutritional level could decrease the bacterioplankton alpha diversity. Considering that beta diversity often produces a beta with a hidden dependence on alpha^46^, the dissimilarity of Bray–Curtis distances was used to indicate the corresponding habitats of bacterioplankton. It can be seen that bacterioplankton communities from DL Reservoir and WL Reservoir significantly differed, as confirmed by the ANOSIM figure and co-occurrence patterns, reflecting the environmental filters in assembling microbial community structures^44^. Considering that WT affects the water–carbonate rock–CO_2_ gas interactions, which in turn can influence trophic status and other hydrochemical factors, the key factor (WT, TN or DOC) shaping karst aquatic bacterioplankton community compositions could not be recognized. Then, karst level, trophic status (including TN and DOC) and hydrochemical factors as combinations of related factor revealed that trophic status exerted significant negative direct effects on bacterioplankton community and diversity, similar to a previous report that nutrition in freshwater reservoirs was the important force for altered bacterial richness and diversity^7,8^. Though a better understanding of their roles in bacterioplankton assemblages is still needed in the future, our study indicated that trophic status was the main factor determining the bacterioplankton distribution and community in karst reservoir, leading to the highlights that environmental filters on bacterioplankton did not act independently^47^.

## Conclusion

In our study, 16S rRNA gene next-generation sequencing coupled with analysis of water chemistry was used to explore the bacterioplankton assemblages in two subtropical karst reservoirs with contrasting trophic status from southwestern China. The results revealed that bacterioplankton communities of the two reservoirs were different. The two reservoirs were dominated by Proteobacteria, Firmicutes, Actinobacteria, Bacteroidetes, Cyanobacteria and Verrucomicrobia with contrasting relative frequency, and it was detected that Proteobacteria and Bacteroidetes were the core communities, which played important roles in karst biogeochemical cycles. The contrasting bacterioplankton assemblages in DL Reservoir and WL Reservoir was determined by trophic status, and environmental factors such as WT, TN and DOC were the decisive factors shaping karst aquatic bacterioplankton. Consequently, our findings provide a baseline for further research about the association between bacterioplankton community and trophic status at a broader scale in karst water systems.

## Supplementary Information

Supplementary Information.

## Data Availability

Raw sequence reads have been deposited to NCBI Sequence Read Archive under the accession number PRJNA495689.

## Abbreviations

DL Reservoir: Dalongdong Reservoir
WL Reservoir: Wulixia Reservoir
DO: Dissolved oxygen
EC: Electrical conductivity
WT: Water temperature
TSI: Trophic state index
GZAR: Guangxi Zhuang Autonomous Region
Chl a: Chlorophyll a
SD: Secchi disc
TP: Total phosphorus
TN: Total nitrogen
TDS: Total dissolved solid
TOC: Total organic carbon
DOC: Dissolved organic carbon
OTU: Operational taxonomic unit
PCoA: Principal coordinates analysis
ANOSIM: Analysis of similarity
RDA: Redundancy analysis
PM: Partial Mantel
PLS-PM: Partial least squares-path model
VPA: Variance partitioning analysis
GoF: Goodness of fit
