# Supplementary material for: Comparative analysis of bacterioplankton assemblages from two subtropical karst reservoirs of southwestern China with contrasting trophic status
Source: Sci Rep. 2020 Dec 18;10:22296. doi: 10.1038/s41598-020-78459-z (PMC7749139; doi:10.1038/s41598-020-78459-z)
Supplement: Supplementary file 1 — Supplementary Information. [file 41598_2020_78459_MOESM1_ESM.docx]

Comparative analysis of bacterioplankton assemblagesfrom two subtropical karst reservoirs of southwestern China with contrasting trophic status

Qiang Li^1,2*^ , Yadan Huang^3^ ,Shenglin Xin^1,2^ , Zhongyi Li^4*^

^1^Key Laboratory of Karst Dynamics, MNR & GZAR, Institute of Karst Geology, Chinese Academy of Geological Sciences, Guilin 541004, China

^2^International Research Center on Karst under the Auspices of UNESCO, Guilin 541004, China

^3^Graduate School of Guilin Medical University, Guilin 541004, China

^4^Agricultural Resource and Environment Research Institute, Guangxi Academy of Agricultural Sciences, Nanning 530007, China

*Correspondence: [glqiangli@hotmail.com](mailto:glqiangli@hotmail.com) and [lizhongyi2007@163.com](mailto:lizhongyi2007@163.com)


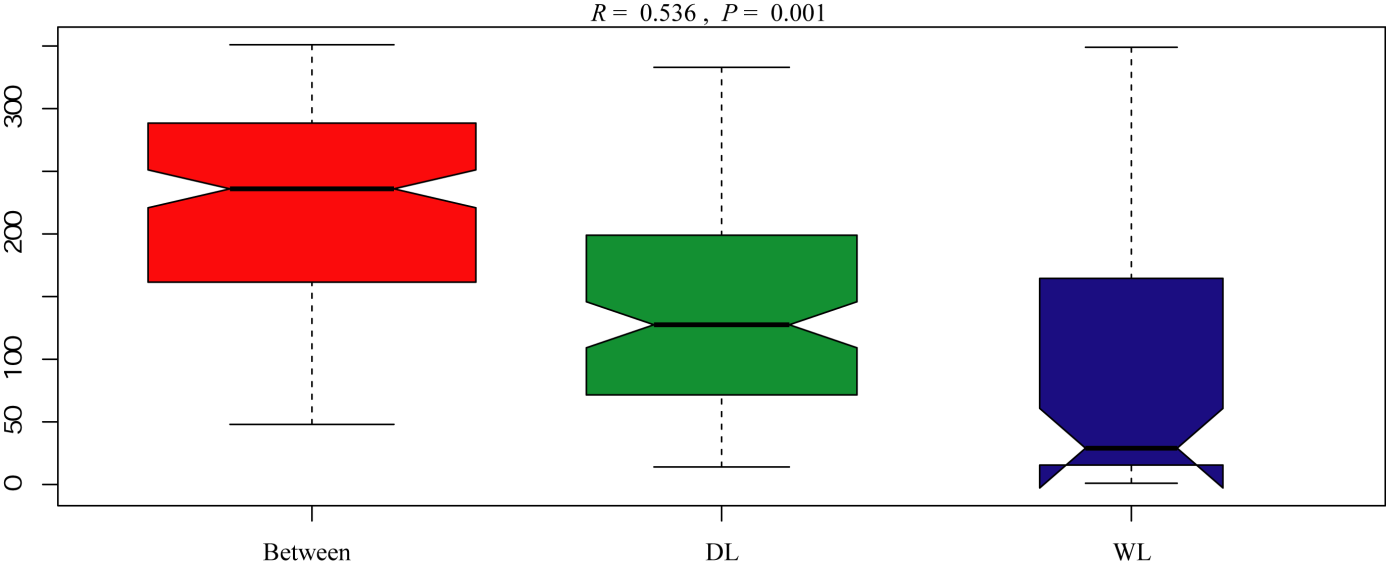


Figure S1 ANOSIM analysis based on the Bray-Curtis distances representing bacterioplankton community dissimilarity between DL Reservoir and WL Reservoir.


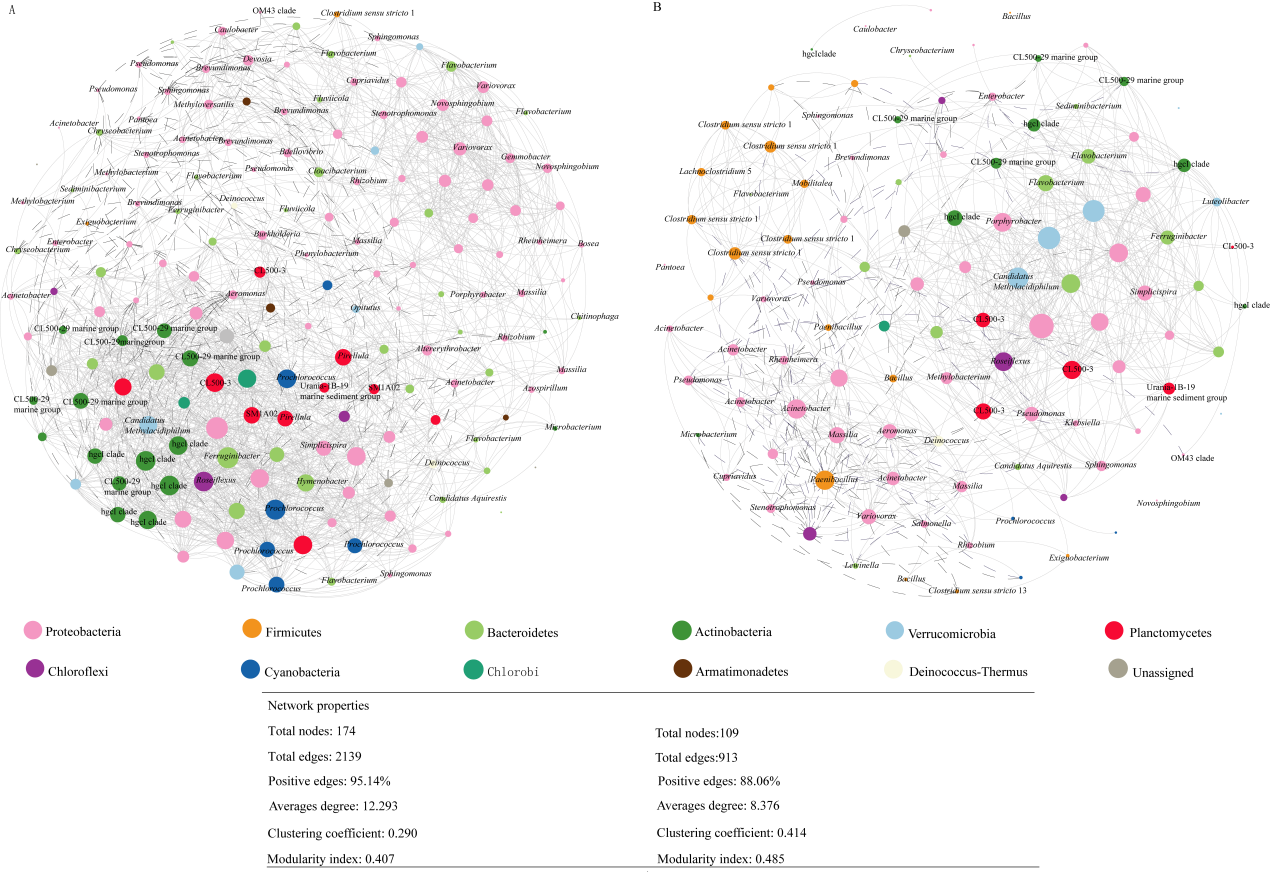


Figure S2 Co-occurrence networks and topological properties of bacterioplankton from WL Reservoir (A) and DL Reservoir (B) based on OTU-level data (relative abundances>0.05%). Nodes are colored according to bacterioplankton phylum and the node size is proportional to their relative frequency at phylum level. OTUs that can be classified at the genus level are marked on the networks. The black dotted lines and gray solid lines represent negative and positive correlations, respectively. Modularity index> 0.4 suggests that the network has a modular structure.


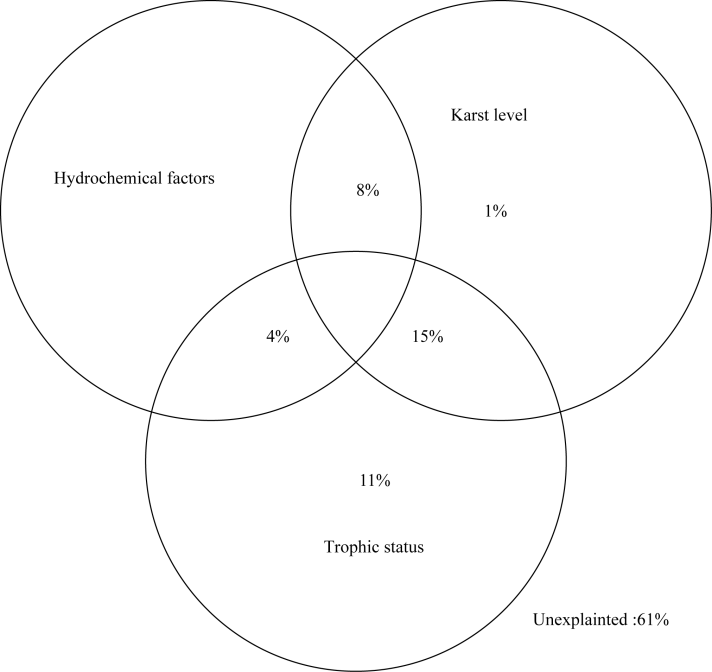


Figure S3 VPA representing the effects of karst level, trophic status and hydrochemical factors on bacterioplankton community. Percentages are the variation of bacterioplankton community explained by karst level, trophic status and hydrochemical factors.
